# Synthesis of New Imidazolidin-2,4-dione and 2-Thioxo-imidazolidin-4-ones *via* *C*-Phenylglycine Derivatives

**DOI:** 10.3390/molecules15010128

**Published:** 2009-12-30

**Authors:** José Alixandre de Sousa Luis, José Maria Barbosa Filho, Bruno Freitas Lira, Isac Almeida Medeiros, Liana Clébia Soares Lima de Morais, Alexsandro Fernandes dos Santos, Cledualdo Soares de Oliveira, Petrônio Filgueiras de Athayde-Filho

**Affiliations:** 1 Laboratório de Tecnologia Farmacêutica, Universidade Federal da Paraíba, João Pessoa - PB, CEP 58.051-970, Brazil; 2 Centro de Educação e Saúde, Unidade Acadêmica de Saúde, Universidade Federal de Campina Grande, Cuité - PB, CEP 58.175-000, Brazil; 3 Departamento de Química, Universidade Federal da Paraíba, Campus I, João Pessoa - PB, CEP 58059-900, Brazil

**Keywords:** hydantoins, *C*-phenylglycine, imidazolidines, 2-thioxoimidazolidine-4-ones

## Abstract

Hydantoins and their derivatives constitute a group of pharmaceutical compounds with anticonvulsant and antiarrhythmic properties, and are also used against diabetes. N-3 and C-5 substituted imidazolidines are examples of such products. As such, we have developed a synthesis of 2,4-dione and 2-thioxo-4-one imidazolidinic derivatives by reaction of amino acids with *C*-phenylglycine, phenyl isocyanate and phenyl isothiocyanate. Four amino-derivatives **IG(1-4)** and eight imidazolidinic derivatives, **IM(1-8)**, were obtained in yields of 70–74%. The mass, infrared, ^1^H and ^13^C-NMR spectra of representative products are discussed.

## Introduction

Nowadays, there is an incessant search for biological functional compounds suitable for treating diverse illnesses. The development of more efficient and less toxic products often involves the study of new synthetic routes or structural modifications of existing molecules and medicinal drugs are often manufactured by modification or molecular variation using bioisosterism [1]. The influence of an atom or an atom group modification by bioisosters can be analyzed based on the biological activity presented by the drug, having an identical or exact antagonistic effect. The synthesis of heterocyclic 2,4-imidazolidinones or hydantoins (1) has been studied intensively for their important pharmacological properties [2]. Substances that contain these heterocyclic moieties present significant biological activities as antifungal [3], antibacterial and anti-inflammatory [4] drugs, for the treatment of hypoglycemia [5], or as plant growth inhibitors [6], among other properties. Thiohydantoins are sulfur analogs of hydantoins with one or two carbonyl groups replaced by thiocarbonyl groups. The 2-thiohydantoins have been widely evaluated due to their applications as hypolipidemic, anti-carcinogenic, antiviral (e.g., herpes virus, HSV, HIV and tuberculosis), antimicrobial, anti-ulcer and anti-inflammatory agents [7].

Several studies [8,9,10] have described the synthesis of amino acid compounds, their importance and applications as intermediates for the synthesis of heterocyclics [11,12]. The present study aimed to contribute with the chemical and pharmacological studies of imidazolidinic compounds, whereby imidazolidinic bioisosters obtained from amino acids were synthesized and characterized as imidazolidin-2,4-dione and 2-thioxo-imidazolidin-4-one derivatives. Among the compounds synthesized we evaluated 3-phenyl-5-(4-isopropylphenyl)-imidazolidin-2,4-dione (**IM-7**), and a focus of this study was to investigate the acute cardiovascular effects induced by **IM-7** in rats. In addition, the effects of 5-(4-ethylphenyl)-3-phenylimidazolidin-2,4-dione (**IM-3**) on the Central Nervous System was investigated, and its possible involvement in antinociception was considered based on results obtained with early pharmacological screening [13]. 

## Results and Discussion

**Scheme 1:**
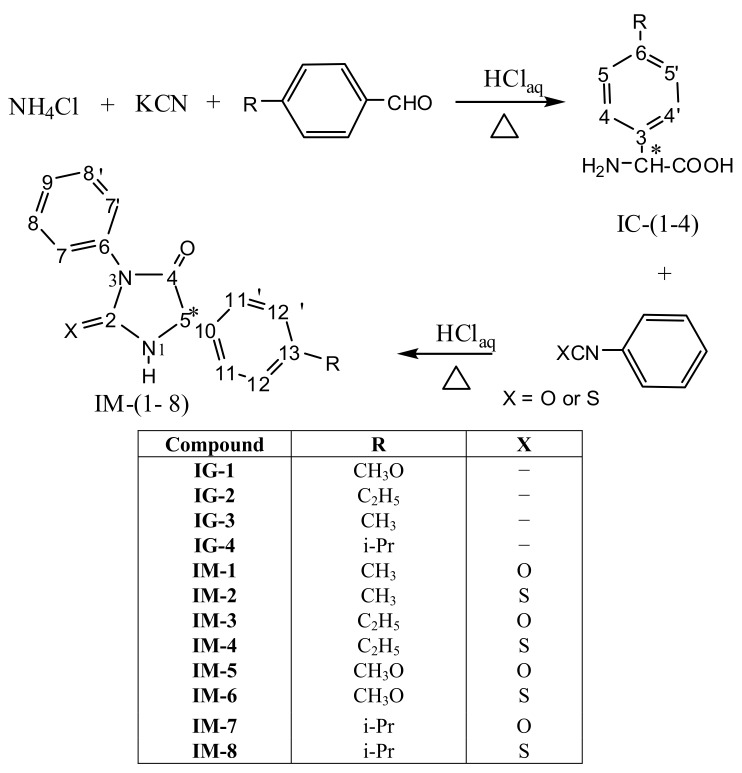
Synthesis of imidazolidines.

Eight 3,5-di-substituted-imidazolidinic compounds were obtained in 70–74% yield by means of amino acids *via* Strecker synthesis. Of these imidazolidinic compounds, one (IM-5) was previously reported [14], but its structure was not elucidated by the usual physical techniques. All samples were obtained in two steps: first, a Strecker synthesis was performed using sodium cyanide, ammonium chloride and 4-arylaldehydes, followed by an acid hydrolysis to form the corresponding *C*-arylglycine derivative (**IG**). *C*-4-Methoxyphenylglycine (**IG-1**), *C*-4-ethylphenylglycine (**IG-2**), *C*-4-methylphenylglycine (**IG-3**) and *C*-4-isopropylphenylglycine (**IG-4**) were thus obtained. In the second part, the amino acids reacted with phenyl isocyanate or phenyl isothiocyanate, followed by an acid hydrolysis reaction (Scheme 1). Eight imidazolidinic compounds (**IM**) were thus obtained: 3-phenyl-5-(4-methylphenyl)-imidazolidin-2,4-dione (**IM-1**); 3-phenyl-5-(4-methylphenyl)-2-thioxoimidazolidin-4-one (**IM-2**); 5-(4-ethylphenyl)-3-phenyl-imidazolidin-2,4-dione (**IM-3**); 3-phenyl-5-(4-ethylphenyl)-2-thioxoimidazolidin-4-one (**IM-4**); 5-(4-methoxyphenyl)-3-phenylimidazolidin-2,4-dione (**IM-5**); 3-phenyl-5-(4-metoxyphenyl)-2-thioxo-imidazolidin-4-one (**IM-6**); 5-(4-isopropylphenyl)-3-phenylimidazolidin-2,4-dione (**IM-7**); 3-phenyl-5-(4-isopropylphenyl)-2-thixo-imidazolidin-4-one (**IM-8**) (Scheme 1).

**Scheme 2:**
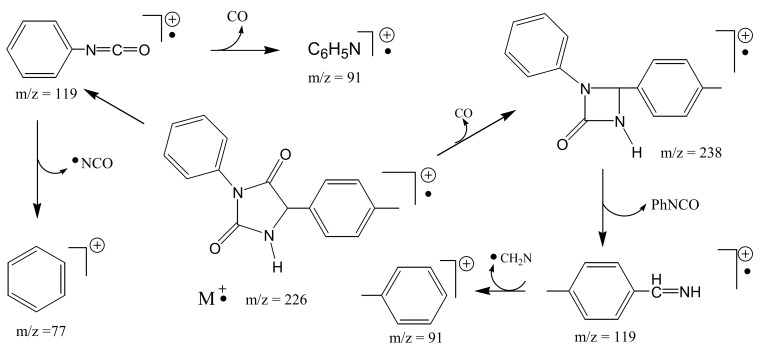
MS fragmentation pattern for the imidazolidines.

Mass spectral data support the proposed structures. Scheme 2 shows the fragmentation pattern for 3-phenyl-5-(4-methylphenyl)-imidazolidin-2,4-dione (**IM-1**) which serves as an example for all the compounds *mutatis mutandis*.

The IR spectra are in agreement with the organic functionalities present. The principal features observed in the intermediates **IG(1-4)** are the carboxylic acid group C=O and C-O^-^ stretches at 1,753–1,740 and 1,422–1,398 cm^-1^, whereas the N-H stretch of the amine group is observed at 3,165–3,100 cm^-1^. Mainly N-H, C=O and C=S stretches were detected in the imidazolidinic rings. The heterocyclic N-H was characterized by the absorption band at 3,317–3,154 cm^–1^. The C=O groups were characterized by absorptions at 1,773 – 1,711 cm^-1^. The C=S absorptions in **IM-2**, **IM-4**, **IM-6** and **IM-8** were characterized by bands at 1,513, 1,518, 1,515 and 1,517 cm^-1^, respectively. The absorption bands at 1,025 and 1,029 cm^-1^ refer the C-O stretch of methoxyl groups of the compounds **IM-5** and **IM-6**. 

The ^1^H-NMR spectra are useful for the determination of structures. In the intermediates **IG-(1-4)** and imidazolidinic compounds **IM(1-8)**, the benzene ring hydrogens show peaks at δ 6.93–7.54 ppm. In **IG(1-4)**, the α-hydrogen signals are at δ 4.83, 4.98, 4.96 and 4.78 ppm. The methoxyl groups in **IG-1**, **IM-5** and **IM-6** is characterized by singlets at 3.72, 3.71 and 3.73 ppm, respectively. The ethylic group in **IG-2**, **IM-3** and **IM-4** is characterized by triplets at 1.07, 1.16 and 1.18 ppm coupled with quartets at 2.53, 2.60 and 2.64 ppm. The methylic groups in **IG-3**, **IM-1** and **IM-2** were characterized by singlets at 2.27, 2.49 and 2.27 ppm. The isopropyl group in **IG-4**, **IM-7** and **IM-8** is characterized by doublets at 0.84, 1.22 and 1.21 ppm, respectively, coupled with septets at 2.18 and 2.90 ppm for the last two compounds. In all imidazolinic compounds the single hydrogen linked to the heterocyclic ring occur as singlet in the range 5.16 – 5.58 ppm and the N-H signals occur as singlets in the range 8.86–10.99 ppm (Table 1). The significant ^13^C-NMR spectra signals are listed in Table 2 and are in agreement with the proposed structures.

The pharmacological studies, *in vivo*, with non-anesthetized rats, **IM-7** (1, 5, 10, 20, 30 mg/kg, i.v.) induced hypotension (-3.6 ± 1.6, -4.2 ± 1.4, -4.4 ± 1.6, -24.6 ± 10.9, -32 ± 9.2 %). Compound IM-7 (20, 30 mg/kg, i.v.) also induced bradycardia (-28 ± 15, -50 ± 15 %). Both responses were completely abolished in rats treated with atropine (2 mg/Kg, i.v.). In mesenteric rings **IM-7** (10^-12^–10^-3^ M) induced relaxation of phenylephrine (10 mM) induced tone (EC_50_ = 2.9 ± 0.4x10^-5^ M). This effect was significantly attenuated after removal of the vascular endothelium, 100 mM L-NAME, or 1 nM atropine (EC_50_ = 1.2 ± 0.1 × 10^-4^; 1.4 ± 0.2 × 10^-4^; 1.3 ± 0.3 × 10^-4^ M, respectively). However, **IM-7** induced relaxant effect was not attenuated by indomethacin (30 mM). 

The present study showed that treatment of mice with 5-(4-ethylphenyl)-3-phenylimidazolidin-2,4-dione **IM-3** in doses of 250 and 500 mg/kg, i.p caused a significant decrease in writhing numbers after administration (7.3 ± 2.3; 3.6 ± 1.7, respectively) in relation to control (22.1 ± 6.0), indicating a antinociceptive effect, however, the central depressant effect was not confirmed because this compound did not increase the response time in the Hot Plate Test in comparison with a standard drug (morphine 6 mg/kg, i.p).

## Experimental

### General

Mass spectra were obtained using a Finnigan GCQ Mat type quadrupole-ion trap spectrometer. IR spectra were obtained by means of a Bruker IFS66 spectrometer with the samples in KBr discs. ^1^H- and ^13^C-NMR spectra were recorded on a Varian Unity Plus 200 MHz spectrometer operating at 200 MHz for ^1^H and 50 MHz for ^13^C, the sample being dissolved in DMSO-d_6_ with TMS as reference. Elemental analysis was carried out using a Perkin Elmer Elemental Microanalyser. The melting points were determined using a Kofler hot-plate apparatus combined with a Carl-Zeiss microscope and are uncorrected. In the pharmacological studies with **IM-7**, male Wistar rats (250–300 g) were anesthetized and the abdominal aorta and inferior vena cava were cannulated for pressure recordings and administration of drugs. Rat superior mesenteric rings (1-2 mm) were suspended by cotton threads for isometric tension recordings in Tyrode´s solution, 37 °C, gassed with 95% O_2_ and 5% CO_2_, resting tension 0.75 g. For the pharmacological studies of **IM-3**, Male Swiss mice (25–35 g) were treated with increased doses of **IM-3** (125, 250 and 500 mg/kg) in the toxicological Test, for calculus of Median Letal Dose (LD_50_), *a posteriori* writhing and Hot Plate Tests were realized. The parameters utilized were writhing numbers, reaction time(s) and latency(s). 

### General method for the preparation of C-arylglycines 

Appropriate amounts of KCN and ammonium chloride were dissolved in distilled H_2_O (100 mL). Equimolar quantities of the arylaldehyde in MeOH (100 mL), were added under vigorous stirring and the reaction continued for 120 minutes. H_2_O (250 mL) was added and the resulting mixture was then added to toluene (250 mL). The toluene phase was separated and then extracted with HCl (6N, 3 × 100 mL). The combined acid extract was refluxed for 8 hours, giving the desired product in the form of white crystals after cooling. These were filtered off, washed with CHCl_3_ and air-dried.

*(±)C-(4-Methoxyphenyl)glycine* (**IG-1**). KCN (24.80 g; 381 mmol), NH_4_Cl (20.38 g; 381 mmol) and 4-methoxybenzaldehyde (50.00 g, 367 mmol), were reacted according to the general procedure. Yield: 70.30% (46.80 g); recrystallization from EtOH/H_2_O (1:1); Mp 230 – 232 °C. IR ν_max_ 3,165 (NH); 2,960 and 2,839 (CH_3_); 1,748 (C=O); 1,413 (C_Ar_-O); 1,028 (CH_3_-O) cm^-1^. ^1^H-NMR δ 3.72 (s, 3H, CH_3_O); 4.83 (s, 1H, H2); 6.93–7.39 (m, 4H, aromatics); 8.92 (s, 2H, NH_2_) ppm. ^13^C-NMR δ 60.0 (CH_3_O); 60.4 (C2); 119.2 (C4-4’); 130.3 (C3); 134.7 (C5-5’); 164.9 (C6); 174.9 (C1).

*(±)C-(4-Ethylphenyl)-glycine* (**IG-2**). KCN (12.37 g; 190 mmol), NH_4_Cl (10.16 g; 190 mmol) and 4-ethylbenzaldehyde (25 g; 186 mmol), were reacted according to the general procedure. Yield: 73.10% (24.40 g); recrystallization from EtOH/H_2_O (1:1); Mp 228–230 °C. IR ν_max_ 3,139 (NH); 2,976 and 2,945 (CH_2_, CH_3_); 1,745 (C=O); 1,422 (C-O) cm^-1^. ^1^H-NMR δ 1.07 (t, 3H, C*H*_3_CH_2_ *J* = 7.6 Hz); 2.53 (q, 2H, CH_3_C*H*_2_ *J* = 7.6 Hz); 4.98 (s, 1H, H2); 7.17–7.33 (m, 4H, aromatics); 8.92 (s, 2H, NH_2_) ppm. ^13^C-NMR δ 15.8 (*C*H_3_CH_2_); 28.1 (CH_3_*C*H_2_); 55.6 (C2); 128.5 (C4-4’ and C5-5’); 130.6 (C3); 145.3 (C6); 169.8 (C1) ppm.

*(±)C-(4-Methylphenyl)-glycine* (**IG-3**). KCN (16.27 g; 250 mmol), NH_4_Cl (13.37 g; 250 mmol) and 4-methylbenzaldehyde (30 g; 250 mmol), were reacted according to the general procedure. Yield: 74.64% (30.80 g); recrystallization from EtOH/H_2_O (1:1); Mp 287 – 289 ^o^C. IR ν_max_ 3,100 (NH); 1,740 (C=O); 1,405 (C-O); 2,967 (CH_3_) cm^-1^. ^1^H-NMR δ 2.27 (s, 3H, CH_3_); 4.96 (s, 1H, H2); 7.21 (d, 2H, H4-4` *J* = 8.2 Hz); 7.39 (d, 2H, H5-5` *J* = 8.2 Hz); 9.02 (s, 2H, NH_2_) ppm. ^13^C-NMR δ 20.9 (CH_3_); 55.3 (C2); 128.2 (C4-4`); 129.4 (C5-5´); 130.4 (C3); 138.8 (C6); 169.8 (C1) ppm.

*(±)C-(4-Isopropylphenyl)-glycine* (**IG-4**). KCN (7.16 g; 110 mmol), NH_4_Cl (5.88 g; 110 mmol) and 4-isopropylbenzaldehyde (16.60 g; 112 mmol), were reacted according to the general procedure. Yield: 72.30% (15.64 g); recrystallization from EtOH/H_2_O (1:1); Mp 182 °C. IR ν_max_ 3,153 (NH); 1,753 (C=O); 1,398 (C-O) cm^-1^. ^1^H-NMR δ 0.84 (d, 6H, CH(CH_3_)_2_, *J* = 6.4 Hz); 2.18 (septet, 1H, CH(CH_3_)_2_, *J* = 6.4 Hz); 4.78 (s, 1H, C*H); 7.01 (d, 2H, aromatics, *J* = 8.0 Hz); 7,11(d, 2H, aromatics, *J* = 8.0 Hz), 9.44 (s, 2H, NH_2_) ppm. ^13^C-NMR δ 23.9 (CH(CH_3_)_2_); 30.9 (CH(CH_3_)_2_); 60.3 (C-2); 127.5 (C-4, 4’); 128.5 (C-3); 128.90 (C-5, 5’); 151.0 (C-6); 170.0 (C-1) ppm.

### General method for the preparation of 3-phenyl-5-arylimidazolidinic derivatives

*C*-Arylglycine was dissolved in the minimum amount of aqueous NaOH (10%) with stirring which was continued for an additional 120 minutes. Equimolar quantities of the required phenyl isocyanate or phenyl isothiocyanate was added in small amounts and stirring continued for a further 4 h. After 24 hours the precipitate was separated by filtration and the remaining solution was acidified with HCl. The aroylimidazilidinic acid obtained was refluxed for 1 h with 40 mL of 6N HCl solution. The white crystalline product was filtered off, washed with H_2_O, air-dried and recrystallized from ethanol/H_2_O (1:1).

*(±)-3-Phenyl-5-(4-methylphenyl)-imidazolidine-2,4-dione* (**IM-1**). *C*-4-Methylphenylglycine (1.48 g; 9 mmol) and PhNCO (1.07 g; 9 mmol), were reacted according to the general procedure. Yield: 77.50% (1.85 g) as white crystals; recrystallization from EtOH/H_2_O (1:1); Mp 198–199 °C. IR ν_max_ 3,236 (NH); 2,921 (CH_3_); 1,715 (C=O) cm^-1^. ^1^H-NMR δ 2.49 (s, 3H, Ar-CH_3_); 5.52 (s, 1H, C5); 7.41–7.71 (m, 9H, aromatics); 9.21 (s, 1H, NH) ppm. ^13^C-NMR δ 21.1 (CH_3_); 60.2 (C5); 127.2 (C12-12´); 127.3 (C11-11´); 128.4 (C9); 129.3 (C8-8´); 129.8 (C7-7´); 132.4 (C10); 133.0 (C6); 138.4 (C13); 156.2 (C4); 172.3 (C2) ppm. EIMS, m/z 266 [M]^⨥^ (41.79); 238 (8.58); 119 (100); 195 (4.34); 103 (2.95); 132 (7.72); 104 (2.27); 77 (8.58); 91 (29.04); 147 (3.40).

*(±)3-Phenyl-5-(4-methylphenyl)-2-thioxo-imidazolidine-4-one* (**IM-2**). *C*-4-Methylphenylglycine (1.65 g; 10 mmol) and PhNCS (1.35 g; 10 mmol), were reacted according to the general procedure. Yield: 78.60% (2.26 g) as white crystals; recrystallization from EtOH/H_2_O (1:1); Mp 215–216 °C. IR ν_max_ 3,154 (NH), 2,986 (CH_3_); 1,759 (C=O); 1,513 (C=S) cm^-1^. ^1^H-NMR δ 2.27 (s, 3H, CH_3_); 5.49 (s, 1H, H5); 7.24–7.47 (m, 9H, aromatics); 10.99 (s, 1H, NH) ppm. ^13^C-NMR δ 21.2 (CH_3_-Ar); 63.1 (C5); 127.4 (C12-12´); 129.2 (C11-11´); 129.3 (C9); 129.4 (C8-8´); 130.1 (C7-7´); 131.7 (C10); 133.7 (C6); 138.9 (C13); 173.4 (C4); 183.1 (C2) ppm. EIMS, m/z 282 [M]^⨥^ (48.8); 254 (7.63); 135 (8.23); 119 (24.13); 103 (4.87); 132 (100); 104 (10.63); 77 (12.73); 91 (13.80); 163 (2.14). 

*(±)3-Phenyl-5-(4-ethyphenyl)-imidazolidine-2,4-dione* (**IM-3**). *C*-4-Ethylphenylglycine (1.61 g; 9 mmol) and PhNCO (1.07 g; 9 mmol), were reacted according to the general procedure. Yield: 78.64% (1.98 g) as white crystals; recrystallization from EtOH/H_2_O (1:1); Mp 216–218 °C. IR ν_max_ 3,241 (NH); 2,966 and 2,930 (CH_2_, CH_3_); 1,773 and 1,711 (C=O) cm^-1^. ^1^H-NMR δ 1.16 (t, 3H, C*H*_3_CH_2_ *J* = 7.6 Hz); 2.60 (q, 2H, CH_3_C*H*_2_ *J* = 7.6 Hz); 5.33 (s, 1H, H5); 7.24-7.51 (m, 9H, aromatics); 8.97 (s, 1H, NH) ppm. ^13^C-NMR δ 15.7 (*C*H_3_CH_2_); 27.9 (CH_3_*C*H_2_); 59.8 (C5); 126.8 (C12-12’); 127.1 (C11-11’); 127.9 (C9); 128.2 (C8-8’); 128.8 (C7-7’); 132.2 (C10); 133.1 (C6); 144.3 (C13); 155.8 (C2); 171.9 (C=O) ppm. EIMS, m/z 280 [M]^⨥^ (47.51); 252 (11.03); 119 (29.75); 209 (9.82); 105 (9.76); 133 (100); 117 (10.28); 146 (2.14); 118 (14.41); 77 (12.49); 91 (40.75); 189 (1.22); 161 (1.97). 

*(±)3-Phenyl-5-(4-ethylphenyl)-2-thioxo-imidazolidine-4-one* (**IM-4**). *C*-4-Ethylphenylglycine (1.61 g; 9 mmol) and PhNCS (1.22 g; 9 mmol), were reacted according to the general procedure. Yield: 73.30 % (1.95 g) as white crystals; recrystallization from EtOH/H_2_O (1:1); Mp 246–248 °C. IR ν_max_ 3,162 (NH); 2,965 and 2,931 (CH_2_, CH_3_). 1,761 (C=O); 1,518 (C=S) cm^-1^. ^1^H-NMR δ 1.18 (t, 3H, C*H*_3_CH_2_, *J* = 7.4 Hz); 2.64 (q, 2H, CH_3_C*H*_2_, *J* = 7.4 Hz); 5.58 (s, 1H, H5); 7.28-7.54 (m, 9H, aromatics); 11.04 (s, 1H, NH) ppm. ^13^C-NMR δ 15.7 (*C*H_3_CH_2_); 28.0 (CH_3_*C*H_2_); 62.6 (C5); 127.1 (C12-12´); 128.4 (C11-11´); 128.7 (C9); 128.8 (C8-8´); 128.9 (C7-7´); 131.7 (C10); 133.4 (C6); 144.6 (C13); 172.9 (C4); 182.7 (C2) ppm. EIMS, m/z 296 [M]^⨥^ (94.29); 268 (5.81); 135 (100); 209 (10.98); 105 (13.61); 133 (33.49); 117 (21.71); 146 (3.33); 118 (13.80); 119 (20.87); 77 (58.80); 91 (38.59); 267 (23.38); 177 (12.76).

*(±)3-Phenyl-5-(4-methoxyphenyl)-imidazolidine-2,4-dione* (**IM-5**). *C*-4-Methoxyphenylglycine (2.54 g; 14 mmol) and PhNCO (1.67 g; 14 mmol), reacted according to the general procedure. Yield: 87.60% (3.47 g) as white crystals; recrystallization from EtOH/H_2_O (1:1); Mp 182–183 °C. IR ν_max_ 3,317 (NH); 1,773, 1,718 (C=O); 1,249 (C_Ar_-O); 1,025 (CH_3_-O) cm^-1^. ^1^H-NMR (DMSO-d_6_) δ 3.71 (s, 3H, CH_3_O); 5.16 (s, 1H, H5); 6.95 (m, 4H, aromatics); 7.32 (m, 5H, aromatics); 8.86 (s, 1H, NH) ppm. ^13^C-NMR (DMSO-d_6_) δ 55.5 (CH_3_O); 59.9 (C5); 114.4 (C12-12’); 128.3 (C9); 128.7 (C8-8’); 129.1 (C7-7’); 129.2 (C11-11’); 130.2 (C10); 140.2 (C6); 154.8 (C2); 159.4 (C13); 173.2 (C4) ppm. EIMS, m/z 282 [M]^⨥^ (32.43); 254 (5.47); 119 (9.27); 211 (2.28); 107 (1.70); 135 (100); 77 (11.43); 91 (9.62); 163 (1.20).

*(±)-3-Phenyl-5-(4-methoxyphenyl)-2-thioxo-imidazolidine-4-one* (**IM-6**). *C*-4-Methoxyphenylglycine (2.54 g; 14 mmol) and PhNCS (1.89 g; 14 mmol), were reacted according to the general procedure. Yield: 85.20% (3.56 g) as white crystals; recrystallization from EtOH/H_2_O (1:1); Mp 226–228 °C. IR ν_max_ 3,154 (NH); 1,717 (C=O); 1,515 (C=S); 1,244 (C_Ar_-O); 1,029 (CH_3_-O) cm^-1^. ^1^H-NMR δ 3.73 (s, 3H, OCH_3_); 5.49 (s, 1H, C5); 7.27-7.51 (m, 9H, aromatics); 10.96 (s, 1H, NH) ppm. ^13^C-NMR δ 55.6 (CH_3_O); 62.7 (C5); 114.8 (C12-12´); 122.8 (C11-11´); 128.8 (C9); 129.2 (C8-8´); 129.3 (C7-7´); 126.5 (C10); 133.6 (C6); 159.9 (C13); 173.5 (C4); 182.9 (C2) ppm. EIMS, m/z 298 [M]^⨥^ (37.65); 135 (60.9) 119 (14.14); 148 (6.71); 77 (22.23); 91 (17.62); 207 (100); 163 (6.32); 267 (9.46). 

*(±)-3-Phenyl-5-(4-isopropylphenyl)-imidazolidine-2,4-dione* (**IM-7**). *C*-4-Isopropylphenylglycine (1.93 g; 10 mmol) and PhNCO (1.19 g; 10 mmol), were reacted according to the general procedure. Yield: 90.80% (2.67 g) as white crystals; recrystallization from EtOH/H_2_O (1:1); Mp 215 °C. IR ν_max_ 3,314 (NH); 1,783 and 1,711(C=O) cm^-1^. ^1^H-NMR δ 1.22 (d, 6H, CH(CH_3_)_2_); 2.90 (septet, 1H, CH(CH_3_)_2_); 5.38 (s, 1H, H5); 7.16 (m, 4H, aromatics); 7.50 (m, 5H, aromatics); 8.97 (s, 1H, NH) ppm. ^13^C-NMR δ 23.9 (CH(CH_3_)_2_); 33.2 (CH(CH_3_)_2_); 59.8 (C5); 126.8 (C12-12’); 127.9 (C9); 127.1 (C8-8’); 126.9 (C7-7’); 128.9 (C11-11’); 132.2 (C10); 133.2 (C6); 155.7 (C2); 148.9 (C13); 171.8 (C4) ppm. EIMS, m/z 294 [M]^⨥^ (54.5); 266(11.5); 147 (76.6); 119 (31.8).

*(±)3-Phenyl-5-(4-isopropylphenyl)-2-thioxo-imidazolidine-4-one* (**IM-8**). *C*-4-Isopropylphenylglycine (1.35 g; 7 mmol) and PhNCS (0.95 g; 7 mmol), were reacted according to the general procedure. Yield: 74.70% (1.62 g) as gray crystals; recrystallization from EtOH/H_2_O (1:1); Mp 255 °C. IR ν_max_ 3,157 (NH); 1,783 (C=O); 1,517 (C=S) cm^-1^. ^1^H-NMR δ 1.21 (d, 6H, CH(CH_3_)_2_); 2.90 (septet, 1H, CH(CH_3_)_2_); 5.55 (s, 1H, H5); 7.28 (m, 4H, aromatics); 7.54 (m, 5H, aromatics); 10.98 (s, 1H, NH) ppm. ^13^C-NMR δ 23.9 (CH(CH_3_)_2_); 33.2 (CH(CH_3_)_2_); 62.6 (C5); 127.0 (C12-12’); 128.7 (C9); 128.8 (C8-8’); 128.9 (C7-7’); 128.7 (C11-11’); 131.8 (C10); 133.4 (C6); 182.7 (C2); 149.2 (C13); 172.9 (C4) ppm. EIMS, m/z 310 [M]^⨥^ (100.0); 297(6.70); 147 (11.5); 120 (6.40); 135 (12.9).

## Conclusions

Four new *C*-phenylglycine derivatives **IG(1-4)** containing different groups and obtained *via* Strecker synthesis were subjected to reactions with phenyl isocyanate and phenyl isothiocyanate to furnish eight imidazolidinic compounds **IM(1-8)**, seven of which were new, and one, **IM-5**, whose structure was not previously elucidated. Their structures were confirmed by infrared, ^1^H- and ^13^C-NMR and mass spectroscopies. The pharmacological studies with **IM-3** and **IM-7** show that these compounds are bioactive structures. In the cardiovascular system **IM-7** induced a marked hypotension and bradycardia which are probably due to decrease of the peripheral resistances. Likewise *in vivo*, the relaxant effect of this compound seems to involve endothelial muscarinic receptor activation and consequent NO release. The results obtained with pharmacological and behavior tests suggest that **IM-3** showed peripheral antinociceptive effect considering the negative results in the Hot Plate test.

## Figures and Tables

**Table 1:** ^1^H-NMR spectra data (DMSO-d_6_): Chemical shifts δ (ppm) from TMS, *J* (Hz).

^1^H	*H-* C*	-C_6_*H*_4_–R	-C_6_*H*_5_	*H*-N	-R
**IG-1, R** = OCH_3_	4.83	6.93 – 7.39 (m, 4H)	-	8.92 (s, 2H)	3.72 (s, 3H)
**IG-2, R** = CH_3_CH_2_	4.98	7.17 – 7.33 (m, 4H)	-	8.92 (s, 2H)	1.07 (t, 3H); 2.53 (q, 2H); *J* = 7.6 Hz
**IG-3, R** = CH_3_	4.96	7.21 (d, 2H); 7.39 (d, 2H), *J* = 8,2 Hz	-	9.02 (s, 2H)	2.27 (s, 3H)
**IG-4, R** = (CH_3_)_2_CH	4.78	7.01 (d, 2H); 7.11 (d, 2H), *J* = 8.0 Hz	-	9.44 (s, 2H)	0.84 (d, 6H); 2.18 (sept, 1H); *J* = 6.4 Hz
**IM-1, R** = CH_3_	5.52 (s, 1H)	7.41 (m, 4H)	7.71 (m, 5H)	9.21 (s, 1H)	2.49 (s, 3H)
**IM-2, R** = CH_3_	5.49 (s, 1H)	7.24 (m, 4H)	7.47 (m, 5H)	10.99 (s, 1H)	2.27 (s, 3H)
**IM-3, R** = CH_3_CH_2_	5.33 (s, 1H)	7.24 (m, 4H)	7.51 (m, 5H)	8.97 (s, 1H)	1.16 (t, 3H); 2.60 (q, 2H); *J* = 7,6 Hz
**IM-4, R** = CH_2_CH_3_	5.58 (s, 1H)	7.28 (m, 4H)	7.54 (m, 5H)	11.04 (s, 1H)	1.18 (t, 3H); 2.64 (q, 2H); *J* = 7,6 Hz
**IM-5, R** = OCH_3_	5.16 (s, 1H)	6.95 (m, 4H)	7.32 (m, 5H)	8.86 (s, 1H)	3.71 (s, 3H)
**IM-6, R** = OCH_3_	5.49 (s, 1H)	7.27 (m, 4H)	7.51 (m, 5H)	10.96 (s,1H)	3.73 (s, 3H)
**IM-7, R** = (CH_3_)_2_CH	5.38 (s, 1H)	7.16 (m, 4H)	7.50 (m, 5H)	8.97 (s, 1H)	1.22 (d, 6H); 2.90 (sept, 1H); *J* = 6.,8 Hz
**IM-8, R** = (CH_3_)_2_CH	5.55 (s, 1H)	7.28 (m, 4H)	7.54 (m, 5H)	10.98 (s, 1H)	1.21 (d, 6H); 2.90 (sept, 1H); *J* = 6,8 Hz

**Table 2:** ^13^C-NMR spectra data (DMSO-d_6_): Chemical shifts δ (ppm) from TMS.

^13^C	C*	C_Ar_-N	C_Ar_-C*	C_Ar_-R	-R	C=S	C=O
**IG-1, R** = OCH_3_	60.4	-	130.3	164.9	60.0	-	174.9
**IG-2, R** = CH_3_CH_2_	55.6	-	130.6	145.3	15.8; 28.1	-	169.8
**IG-3, R** = CH_3_	55.3	-	130.4	138.8	20.9	-	169.8
**IG-4, R** = (CH_3_)_2_CH	60.3	-	128.5	151.0	23.9; 30.9	-	170.0
**IM-1, R** = CH_3_	60.2	132.4	133.0	138,4	21.1	-	156.2; 172.3
**IM-2, R** = CH_3_	63.1	131.7	133.7	138,9	21.2	183.1	173.4
**IM-3, R** = CH_3_CH_2_	59.8	133.1	132.2	144,3	15.7; 27.9	-	155.8; 171.9
**IM-4, R** = CH_3_CH_2_	62.6	131.7	133.4	144,6	15.7; 28.0	182.7	172.9
**IM-5, R** = OCH_3_	59.9	130.2	130.2	159,4	55.5	-	154.8; 173.2
**IM-6, R** = OCH_3_	62.7	133,6	133.6	159,9	55.6	182.9	173.5
**IM-7, R** = (CH_3_)_2_CH	59.8	132.2	133.2	148,9	23.9; 33.2	-	155.7; 171.8
**IM-8, R** = (CH_3_)_2_CH	62.6	131.8	133.4	149,2	23.9; 33.2	182.7	172.9

## References

[1] Cramer R.D., Clark R.D., Patterson D.E., Ferguson A.M. (1996). Bioisosterism as a molecular diversity descriptor: Steric fields of single “topomeric” conformers. J. Med. Chem..

[2] Bateman J.H. (1980). Hydantoin and derivatives. Grayson; Martin; Eckroth, Kirk-Othmer Encyclopedia of Chemical Technology.

[3] Dolezel J., Hirsova P., Opletalova V., Dohnal J., Marcela V., Kunes J., Jampilek J. (2009). Rhodanineacetic acid derivatives as potential drugs:preparation, hydrophobic properties and antifungal activity of (5-arylalkylidene-4-oxo-2-thioxo-1,3-thiazolidin-3-yl)acetic acids. Molecules.

[4] Menezes E.H.C., Góes A.J.S., Diu M.B.S., Galdino S.L., Pitta I.R., Luu-Duc C. (1992). Synthesis and structure of substituted benzyl imidazolidinedione and chlorobenzyl thiazolidine-dione compounds. Pharmazie.

[5] Momose Y., Maekawa T., Yamano T., Kawada M., Odaka H., Ikeda H., Sohda T. (2002). Novel 5-substituted 2,4-thiazolidinedione and 2,4-oxazolidinedione derivatives as insulin sensitizers with antidiabetic activities. J. Med. Chem..

[6] Inamori Y., Muro C., Tanaka R., Adachi A., Miyamoto K., Tsujibo H. (1992). Phytogrowthinhibitory activity of sulfur-containing compounds. I. Inhibitory activities of thiazolidine derivatives on lant growth. Chem. Pharm. Bull..

[7] Wang Z.D., Sheikh S.O., Zhang Y. (2006). A Simple Synthesis of 2-Thiohydantoins. Molecules.

[8] Lira B.F., Athayde-Filho P.F., Miller J., Simas A.M., Dias A.F., Vieira M.J. (2002). Synthesis and characterization of some new mesoionic 1,3-thiazolium-5-thiolates *via* cyclodehydration and in situ 1,3-dipolar cycloaddition/cycloreversion. Molecules.

[9] De Athayde-Filho P.F., Miller J., Simas A.M., Lira B.F., De Souza Luis J.A., Zuckerman-Schpector J. (2003). Synthesis, characterization and crystallographic studies of three 2-aryl-3-methyl-4-aryl-1,3-thiazolium-5-thiolates. Synthesis (Stuttgart).

[10] Lira B.F., Miller J., Simas A.M., Athayde-Filho P.F., Dias A.F., Silva R.O., Oliveira V.C. (2004). Synthesis and complete assignments of 1H and 13C NMR spectra of mesoionic 2-(p-trifluoromethylphenyl)-3-methyl-4-(p-tolyl)-1,3-thiazolium-5-thiolate and 2-(p-chlorophenyl)-3-methyl–4-(p-isopropylphenyl)-1,3-thiazolium-5-thiolate. ARKIVOC.

[11] Bosco C.A.C., Maciel G.S., Rakov N., de Araújo C.B., Acioli L.H., Simas A.M., Athayde-Filho P.F., Miller J. (2007). Probing the nuclear susceptibility of mesoionic compounds using two-beam coupling with chirp-controlled pulses. Chem. Phys. Lett..

[12] Pilla V., de Araújo C.B., Lira B.F., Simas A.M., Miller J., Athayde-Filho P.F. (2006). Nonlinear absorption of new mesoionic compounds. Opt. Commun..

[13] Anjos R.M. (2006). Cardiovascular action of 5-(4-isopropylphenyl)-3-phenyl-imidazolidine-2,4-dione in rats—Approach *in vivo* and *in vitro*. PhD Thesis.

[14] Harvill E.K., Herbst R.M. (1944). The transamination reaction. The effect of various nuclear substituted phenylaminoacetic acids on the course of the reaction. J. Org. Chem..

