# De Sousa Luis, J.A., *et al*. Synthesis of New Imidazolidin-2,4-dione and 2-Thioxo-imidazolidin-4-ones *via* C-Phenylglycine Derivatives. *Molecules* 2010, *15*, 128-137

**DOI:** 10.3390/molecules15031985

**Published:** 2010-03-22

**Authors:** José Alixandre de Sousa Luis, José Maria Barbosa Filho, Bruno Freitas Lira, Isac Almeida Medeiros, Liana Clébia Soares Lima de Morais, Raline Mendonça dos Anjos, Alexsandro Fernandes dos Santos, Cledualdo Soares de Oliveira, Petrônio Filgueiras de Athayde-Filho

**Affiliations:** 1 Laboratório de Tecnologia Farmacêutica, Universidade Federal da Paraíba, João Pessoa - PB, CEP 58.051-970, Brazil; 2 Centro de Educação e Saúde, Unidade Acadêmica de Saúde, Universidade Federal de Campina Grande, Cuité - PB, CEP 58.175-000, Brazil; 3 Departamento de Química, Universidade Federal da Paraíba, Campus I, João Pessoa - PB, CEP 58059-900, Brazil

The authors wish to make the following correction to their paper [1], published recently in *Molecules*. The coauthor Raline Mendonça dos Anjos was omitted from the author list, which should read as follows: 

José Alixandre de Sousa Luis ^1,2^, José Maria Barbosa Filho ^1^, Bruno Freitas Lira ^1^, Isac Almeida Medeiros ^1^, Liana Clébia Soares Lima de Morais ^1^, Raline Mendonça dos Anjos ^1^, Alexsandro Fernandes dos Santos ^3^, Cledualdo Soares de Oliveira ^3^ and Petrônio Filgueiras de Athayde-Filho ^1,3,^*
